# Effect of Cu as a Minority Alloying Element on the Corrosion Behaviour of Amorphous and Crystalline Al-Ni-Si Alloy

**DOI:** 10.3390/ma16155446

**Published:** 2023-08-03

**Authors:** Vanya Dyakova, Yoanna Kostova, Boriana Tzaneva, Hristina Spasova, Daniela Kovacheva

**Affiliations:** 1 Institute of Metal Science, Equipment and Technologies with Hydro- and Aerodynamics Centre “Acad. A. Balevski”, Bulgarian Academy of Sciences, Shipchenski Prohod Str., 67, 1574 Sofia, Bulgaria; y_kostova@is.bas.bg (Y.K.); h.spasova@ims.bas.bg (H.S.); 2 Department of Chemistry, Technical University of Sofia, Kliment Ohridski Blvd., 8, 1000 Sofia, Bulgaria; borianatz@tu-sofia.bg; 3 Institute of General and Inorganic Chemistry, Bulgarian Academy of Sciences, Acad. Angel Bonchev Str., 11, 1113 Sofia, Bulgaria; didka@svr.igic.bas.bg

**Keywords:** corrosion, amorphous Al-Ni-Si alloys, crystallisation, copper additive

## Abstract

The effect of copper as a minority alloying element on the corrosion behaviour of amorphous and crystalline Al_74_Ni_16_Si_10_ and Al_74_Ni_15_Si_9_Cu_2_ alloys was investigated in this study. Amorphous alloys were produced as rapidly solidified ribbons using the Chill Block Melt Spinning (CBMS) method and subsequently annealed to complete crystallisation. The corrosion rate of alloys was obtained through continuous immersion tests in 3.5% NaCl at 25 °C and 50 °C for 360 h. The electrochemical parameters corrosion current density (*J_corr_*) and corrosion potential (*E_corr_*) were determined via the potentiodynamic polarisation test. The results showed better corrosion characteristics of amorphous alloys. The addition of 2 at.% copper to the Al_74_Ni_16_Si_10_ alloy improved pitting corrosion resistance without significant effect on the corrosion current and potential. In immersion tests at 25 °C, the presence of copper resulted in an increase in the corrosion rate of about 300% for both amorphous and crystalline alloys. At a temperature of 50 °C, this increase is on average 130%. The apparent difference between the results of the two test methods is discussed in terms of the imperfections on the surface of rapidly solidified ribbons. The results of this study will contribute to a more complex understanding of the nature of amorphous alloys and their application.

## 1. Introduction

The corrosion resistance of amorphous and crystalline alloys is one of the determining factors for their application. The corrosion properties of amorphous alloys are expected to surpass those of their crystalline analogues for several reasons: (i) the formation of a monophasic solid solution with good chemical and structural homogeneity which eliminates the influence of microstructural defects (including grain boundaries) and particles of a second phase; (ii) the addition of rare earth, transition and refractory elements which stimulate the formation of a passive surface layer with these metals; and (iii) the tendency to repassivation which has been observed in amorphous alloys after the occurrence of local corrosion [1,2].

The first evidence of high corrosion resistance of metallic glasses was reported by Naka, Hashimoto and Masumoto in 1974 [3]. Since then, numerous studies have been published on the corrosion behaviour of amorphous alloys based on iron [4,5,6,7,8,9], zirconium [10,11,12,13], magnesium [14,15,16], copper [17,18,19], aluminium [20,21,22,23,24,25,26,27,28,29,30] and others [31,32,33].

Amorphous and nanocrystalline alloys based on aluminium, especially compositions with transition and rare earth metals, show improved mechanical properties and good corrosion resistance [21]. Vitrification of aluminium alloys via rapid solidification is known to be a difficult process and vitrification of only local areas of the specimens has been found in some cases. All the advantages of homogeneous structure could potentially be lost in the presence of crystal areas. Nevertheless, the corrosion behaviour of rapidly solidified aluminium alloys is of interest even in cases when complete vitrification is not achieved [22].

The research on the influence of minority alloying elements usually focuses on their role in improving the glass formation of metallic glasses. The role of minority solutes in corrosion resistance has not been extensively investigated and is not well understood due to the difficulty of glass formation and the impossibility to produce amorphous alloys in the absence of a minority solute [34].

Zhang et al. studied the influence of Ti and Cr as minority alloying elements on the corrosion behaviour of Al-Ni-Y amorphous alloys [23]. They found that the improved corrosion resistance is due to an increased concentration of Ti or Cr in the formed surface passive films. Furthermore, the effect of Ti is stronger due to the higher Ti concentration in the film, leading to higher film resistance. The effect of Ti and Cr as alloying elements on the corrosion behaviour of amorphous alloys Al-Ti-Mg, Al-Cr-Mo and Al-Cr-Mo was also investigated by Akiyama [24]. The influence of alloying additions on the composition and structure of the passive film was studied. The authors [35] believe that the protective ability of the passive film in aluminium–manganese–molybdenum (Al-Mn-Mo) alloys is due to the formation of a less defective and a more compact structure. They explain the role of the alloying additive Mo in both the substrate and the passive film.

Amorphous aluminium alloys containing W and Mo have been reported to show significant corrosion resistance, especially in chloride environments [25].

Yoshioka et al. [26] investigated the effect of alloying on the pitting corrosion resistance of rapidly solidified binary and ternary aluminium alloys containing Mg, Ti, Mn, Cr, Fe, Ni, Cu, Zn, Zr, and Nb. They proved that the rapid melt spinning method is quite effective in refining the pitting potential by several hundred millivolts for all alloys, except those containing Mg, Fe, or Zn.

The influence of the transition metals ratio on the corrosion electrochemical behaviour of Al–Ni–Co–Nd amorphous alloy was investigated by Karfidov et al. [27]. According to the gravimetric and chemical analysis data, the Al_86_Ni_4_Co_4_Nd_6_ alloy was found to demonstrate higher corrosion resistance than Al_86_Ni_6_Co_2_Nd_6_. With the increase in Ni and decrease in Co concentrations, the corrosion potential shifted in an electronegative direction. All amorphous compositions investigated in [27] had a wide passivation area due to the formation of a stable layer of aluminium oxide on the surface, and this determined their high corrosion resistance.

Aburada et al. [28] reported on the positive influence of Ni as a minority alloying element (Ni < 2 at.%) on the corrosion resistance of amorphous (Al_75_Cu_19_Mg_6_)_100−x_Ni_x_ alloys. Nickel was considered superior to Cu for improving the pitting resistance of Al.

Research on the effect of copper on the corrosion behaviour of aluminium alloys is incomplete and the results are conflicting The addition of small amounts of copper (about 1.5%) is known to increase the strength and hardness of commercial Al-based alloys [36,37]. In a NaCl solution, when copper content increases (within the solubility limit of Cu in Al), the current density is reduced [38] and the pitting potential shifts in the positive direction [39]. In contrast, Wang et al. demonstrated that in the case of prolonged salt spray exposure to Al-Si-Mg-Cr alloys, copper deteriorates the corrosion resistance [40]. Deterioration of corrosion resistance is mainly associated with the separation of secondary Cu-rich phases, which have cathode relations concerning the aluminium matrix during the pit initiation [41].

In our previous research, we investigated the influence of Ni on the corrosion behaviour of amorphous alloys (Al_74_Cu_16_Mg_10_)_100−x_Ni_x_ (x = 1, 2, 3 at.%) [29]. The results showed a significant improvement in the pitting corrosion resistance of all amorphous alloys compared to their crystalline analogues, which was explained by the absence of secondary phases Al_2_Cu and Al_2_CuMg in the chemically homogenous amorphous structure.

The Influence of minority alloying elements, Zn and Zr, on the corrosion behaviour of rapidly solidified and annealed nanocrysalline Al_74_Cu_16_Mg_10_ alloys was studied in [30]. The effect of the amorphous–crystalline structure transformation on the corrosion rate was negative, and the effect was most significant in the Zn-containing alloy. The main reason for the registered enhanced local corrosion in the crystalline alloys was the chemical and structural inhomogeneity due to the presence of the active intermetallic phases, Al_2_CuMg, Al_2_(Cu,Zn), and Al_3_Zr_4_, in the aluminium matrix.

Yoshioka et al. [26] studied the corrosion behaviour of conventionally cast and rapidly solidified Al-Ni-Si alloys. They reported that in conventionally cast Al-Ni-Si alloys, pitting corrosion usually attacked the Al_3_Ni phase. Rapidly solidified alloys formed an α-Al phase supersaturated with dissolved Ni and Si atoms, and this resulted in a reduced amount of silicon phases and intermetallic compounds, which are usually responsible for preferential corrosion attack.

Gao et al. [42] synthesised and investigated dense Al-based amorphous metallic coating deposited on AA 2024 substrate. They produced the coating using high-velocity air fuel (HVAF) spraying of an Al_86_Ni_6_Y_4.5_Co_2_La_1.5_ alloy which is known to possess high glass-forming ability. The authors found an enhanced corrosion resistance of the coating compared to AA 2024, which provided a sacrificial anode-based cathodic protection for the substrate under a chloride-containing environment.

Most available studies confirm a better corrosion resistance of amorphous glasses compared to their crystalline analogues [39], but in some cases, no improved corrosion resistance has been found in amorphous alloys, despite their homogeneous and defect-free microstructure [43,44,45,46]. It is also not yet clear how the level of partial crystallisation affects the corrosion resistance, what is the effect of the minor surface imperfections, what is the influence of the test method and duration, etc. The influence of the material surface, as the interface between the material and the corrosive environment, is an important factor in evaluating the corrosion behaviour of amorphous metals [47]. Despite the lack of boundaries, segregation or other defects in the microstructure of the corrosion process is influenced by both the roughness and the chemical inhomogeneity of metallic glasses. Unfortunately, these topics are hardly addressed in scientific research. The topic of corrosion mechanisms in different types of metallic materials is also discussed in a limited number of scientific studies [48].

In our previous studies, we investigated the effect of copper as a minority alloying element on the glass formation of a nearly eutectic Al-Ni-Si alloy. There is a lack of data in the scientific literature on the effect of small amounts of Cu on the corrosion properties of amorphous Al-based alloys. This study aims to evaluate the influence of 2 at.% copper on the corrosion resistance of amorphous Al-Ni-Si alloys and their crystalline analogues. We believe that this will contribute in enriching the knowledge of the influence of the amorphous–crystalline transition on the properties of Al-Ni-Si and Al-Ni-Si-Cu alloys.

## 2. Materials and Methods

### 2.1. Materials

Al-Ni-Si and Al-Ni-Si-Cu alloys were synthesised from pure metals of Al, Ni, Si and Cu in an installation including a resistance electric furnace mounted in a water-cooled pneumovacuum chamber in an argon atmosphere. The alloys were obtained in the form of ribbons using the Chill Block Melt Spinning (CBMS) method. They are about 3–4 mm wide and 26–40 µm thick. To obtain a crystalline structure, the ribbons were annealed at a temperature of 350 °C, which is 100 °C higher than their crystallisation temperatures determined via the DSC analysis. The production processes of the ligatures and the ribbons were described in detail in our previous publications [49].

The composition of the alloy was determined based on our preliminary studies, which showed that a near-eutectic composition is optimal for producing an amorphous Al-Ni-Si alloy. We determined the amount of copper added to be 2 at.% based on research to combine good GFA, mechanical properties and corrosion resistance [50,51,52].

The chemical composition of the Al-Ni-Si and Al-Ni-Si-Cu ribbons was determined using a HIROX 5500 scanning electron microscope (SEM, HIROX Europe, Limonest, France) with a BRUCKER EXDS system (BRUCKER Co., Germany). The average values of the results obtained from measurements in 15 fields are presented in Table 1. The indices in the designation of the alloys correspond to the content of the elements Al, Ni, Si and Cu in at.%. Based on the obtained results, the designation of the Al_-_Ni_-_Si and Al_-_Ni_-_Si_-_Cu alloys is Al_74_Ni_16_Si_10_ and Al_74_Ni_15_Si_9_Cu_9_, respectively.

### 2.2. Characterisation Methods

The microstructure of the rapidly solidified and annealed alloys was characterised using X-ray diffraction analysis (XRD) and transmission electron microscopy (TEM) observation and electron diffraction.

XRD analysis was carried out for determining the phase composition of the alloys and corrosion products. A Bruker D8 Advance powder X-ray diffractometer (Karlsruhe, Germany) with Ni-filtered Cu Kα radiation and LynxEye solid-state position-sensitive detector was applied. The PDF-2 (2021) database of the International Center for Data Diffraction (ICDD) and the DiffracPlusEVA software v.4.0 (Bruker AXS 2010-2014, Karlsruhe, Germany) package was used to perform the phase analysis

XRD proved that the microstructure of the rapidly solidified ribbons Al_74_Ni_16_Si_10_ and Al_74_Ni_15_Si_9_Cu_2_ is completely amorphous. These alloys will be denoted hereafter with the index “am”. The type, amount and sizes of the crystalline phases in the annealed samples were determined. Three phases were registered in the crystalline Al_74_Ni_16_Si_10_ alloy, Al-fcc, Al_3_Ni, and NiSi_2_, with sizes 88 nm, 71 nm, and 43 nm, respectively; in the crystalline Al_74_Ni_16_Si_9_Cu_2_ alloy, four phases were registered, Al-fcc, Al_3_Ni, NiSi_2_, and Cu_3.8_Ni with sizes 136 nm, 88 nm, 45 nm, and 60 nm, respectively and Al_0.4_Cu_0.6_Ni_3_ traces [53]. Based on the XRD results, after devitrification, we designated the annealed Al_74_Ni_16_Si_10_ alloy as a nanocrystalline and the annealed Al_74_Ni_15_Si_9_Cu_2_ alloy as an ultra-fine crystalline. Further in this work, we use the indexes “ncr” and “ufcr”, respectively, for these two alloys [54].

High-Resolution Transmission Electron Microscope (HRTEM) JEOL JEM 2100, (JEOL Ltd., Tokyo, Japan) at an accelerating voltage of 200 kV in Selected Area Diffraction (SAED) and High Resolution (HRTEM) modes was used for transmission electron microscopy observation of the microstructures of the Al-Ni-Si and Al-Ni-Si-Cu amorphous alloys. The results of the observations and electron diffraction confirmed the amorphous nature of the ribbons obtained.

The corrosion products deposited after the immersion test were observed using SEM HIROX 5500 (HIROX Europe, Limonest, France) and were analysed via XRD.

### 2.3. Corrosion Test Methods

Two methods for determining the corrosion properties of Al_74_Ni_16_Si_10_ and Al_74_Ni_15_Si_9_Cu_2_ alloys were applied: gravimetric (immersion) test for testing metals’ resistance to general corrosion and electrochemical polarisation test to indicate the propensity to local corrosion.

General corrosion tests were performed through continuous immersion of specimens in 3.5% NaCl for 360 h in a laboratory thermostat at a temperature of 25 °C and for 50 h at a temperature of 50 °C. Before testing, the specimens were degreased in acetone and treated in diluted HNO_3_ for one minute. The test temperature of 25 °C is the room temperature normally used in standard immersion tests and in electrochemical polarisation tests according to standards ASTM G31 and ASTM G3.

The following formula was used to determine the corrosion rate through the gravimetric tests: CR = $\frac{W}{S.T}$ [g m^−2^h^−1^], where W represents the mass loss index in [g] as the difference between the weight of test specimens before the test and the removal of corrosion products after the test, W = w_1_ − w_2_; S is the area of the specimen [m^2^]; and T is the time of immersion in the corrosion medium [h]. The mass of the samples was weighed on an analytical balance. All CR results obtained are averages of a minimum of three parallel tests [53].

After the test, the test specimens were rinsed with distilled water repeatedly and the released corrosion products were analysed via XRD.

**Figure 1:**
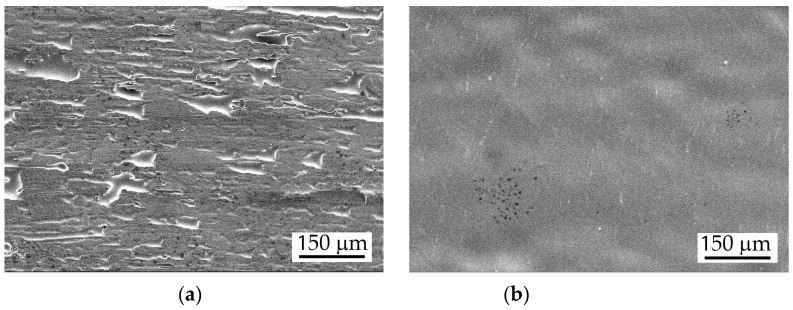
Surface morphology of the amorphous Al_74_Ni_15_Si_9_Cu_2_-am ribbon, SEM: (**a**) surface on the side of cooling copper disk; and (**b**) top surface.

The general and localised corrosion of the ribbons was studied using the cyclic potentiodynamic polarisation method. Test specimens were degreased in alcohol and immersed in a solution of 3.5% NaCl at a temperature of 25 °C. A conventional tri-electrode cell with a working electrode from the studied amorphous and crystalline tested alloys, a Pt plate counter electrode and a silver chloride reference electrode (Ag/AgCl) was used. For the electrochemical test, the surface on the cooling copper disk side (Figure 1a) of ribbons is insulated with varnish to define the working surface at 0.5 cm^2^ on the smoother side (Figure 1b). All potentials in this work reported are relative to the Ag/AgCl electrode. The electrochemical tests were performed with galvanostat-potentiostat Autolab model PGSTAT 204 and computer software NOVA 2.1 (Metrohm Autolab, Netherland, Utrecht).

The specimens were soaked for 10 min in 3.5% NaCl to stabilise the open circuit potential (OCP). Electrochemical impedance spectroscopy (EIS) or potentiodynamic tests were then performed. EIS was carried out at an OSP whit AC potential fluctuations with an amplitude of 10 mV and frequencies from 1 kHz to 0.1 Hz. The cyclic potentiodynamic studies were carried out at a scan rate of 1 mV s^−1^ in an anodic direction from an initial potential of −0.25 V vs. OCP until exceeding the threshold current density of 1 mA cm^−2^; then, the potential was scanned in a reverse cathodic direction until the two branches of the polarisation curve intersect. For each tested alloy, at least 5 specimens were tested to verify the reproducibility of the results.

## 3. Results

### 3.1. Gravimetric Studies

The results of the gravimetric tests for the determination of the corrosion rate CR are presented in Table 2. Based on the averaged values of CR at both test temperatures, the ratios A, B, C, and D were calculated. The ratios A = CR_cr25_/CR_am25_ and B = CR_cr50_/CR_am50_ demonstrate the change in corrosion rate for each alloy after the amorphous → crystalline transformation caused by annealing at the corrosion test temperature of 25 °C or 50 °C, respectively. Analogically, the ratios C=CR_am50_/CR_am25_ and D=CR_cr50_/CR_cr25_ indicate the influence of the test temperature on the corrosion rate in both states, amorphous and nano-/ultra-fine crystalline.

At a test temperature of 25 °C (Table 2), the amorphous Al_74_Ni_16_Si_10_-am alloy has the lowest corrosion rate, CR_am25_. The corrosion rate CR_25_ of the Cu-containing amorphous alloy Al_74_Ni_15_Si_9_Cu_2_-am is about 2.6 times higher compared to the copper-free Al_74_Ni_16_Si_10_-am alloy. At a test temperature of 50 °C, the lowest corrosion rate CR_50_ is obtained again for the amorphous Al_74_Ni_16_Si_10_-am, but its difference with Al_74_Ni_15_Si_9_Cu_2_-am is only about 1.4 times. Nevertheless, the corrosion rates of both amorphous alloys at both temperatures remain low and do not exceed 1 × 10^−2^ g m^−2^h^−1^.

Both annealed alloys, in which the structure was transformed from amorphous to crystalline, have significantly higher corrosion rates at 25 °C and especially at 50 °C than their amorphous analogues. The highest CR50 = 7.3 × 10^−2^ g m^−2^h^−1^ was measured for the ultra-fine crystalline Al_74_Ni_15_Si_9_Cu_2_-ufcr alloy.

It should be noted that at a test temperature of 25 °C, the effect of amorphous → crystalline transformation on the corrosion process is more negative for the Al_74_Ni_15_Si_9_Cu_2_ alloy than for Al_74_Ni_16_Si_10_; the corrosion rate of Al_74_Ni_15_Si_9_Cu_2_-ufcr increases 3.3 times compared to 2.9 times for Al_74_Ni_16_Si_10_-ncr. A reverse dependence is observed at 50 °C; the effect is more negative for Al_74_Ni_16_Si_10_ (B = 8.4) than for Al_74_Ni_15_Si_9_Cu_2_ (B = 7.7). However, taking into account the relatively small deviations (3.3 vs.2.9 for A and 8.4 vs.7.7 for B) and the uncertainty of the tests, it can be assumed that at each of the two test temperatures, the effect of crystallisation is negative and similar for both alloys; the increase in corrosion rate in crystallised alloys at a test temperature of 25 °C is about 3 times and at a test temperature of 50 °C about 8 times.

The increase in test temperature from 25 °C to 50 °C doubled the corrosion rate of the amorphous alloy Al_74_Ni_16_Si_10_-am (C = 1.9), but hardly affected the corrosion rate of the amorphous alloy Al_74_Ni_15_Si_9_Cu_2_-am (C = 1). The tendency for an increase in the corrosion rate of Al_74_Ni_16_Si_10_ with increasing test temperature is observed in the crystalline state also: for Al_74_Ni_16_Si_10_-ncr, it is D = 5.5 ad and for Al_74_Ni_15_Si_9_Cu_2_-ufcr, it is D = 2.4.

**Figure 2:**
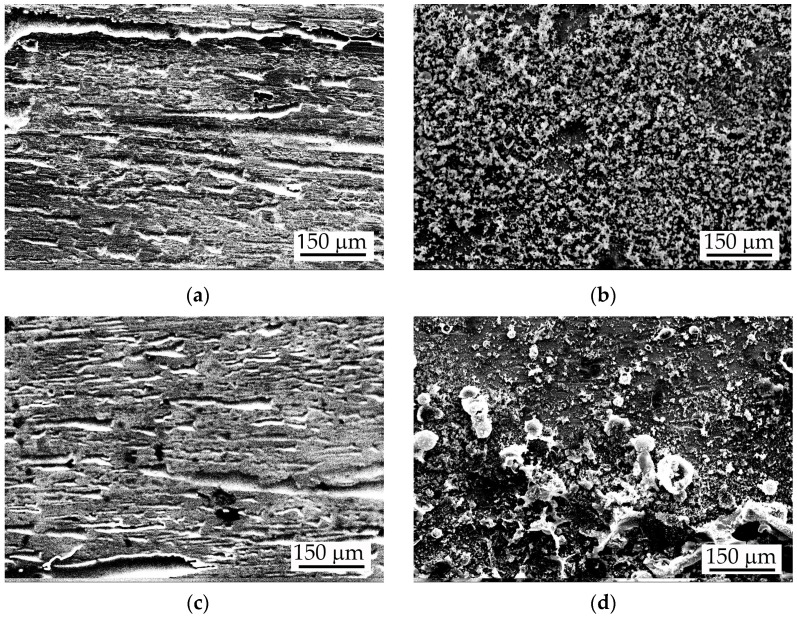
SEM images of Al-Ni-Si and Al-Ni-Si-Cu alloys corroded samples after prolonged immersion test in 3.5% NaCl solution: (**a**) Al_74_Ni_16_Si_10_-am; (**b**) Al_74_Ni_16_Si_10_-ncr; (**c**) Al_74_Ni_15_Si_9_Cu_2_-am; and (**d**) Al_74_Ni_15_Si_9_Cu_2_-ufcr.

**Figure 3:**
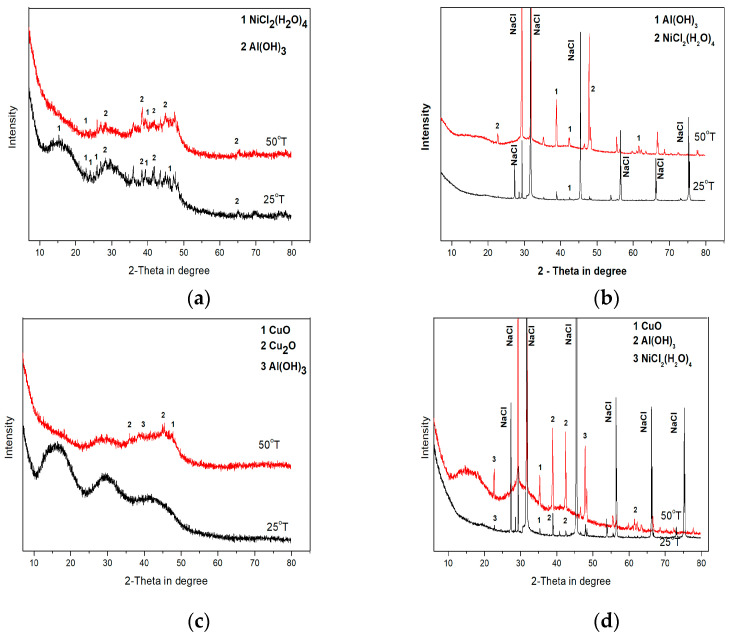
XRD patterns of the corrosion products of Al_74_Ni_16_Si_10_ and Al_74_Ni_15_Si_9_Cu_2_ alloys after 360 h immersion test in 3.5% NaCl; (**a**) Al_74_Ni_16_Si_10_-am; (**b**) Al_74_Ni_16_Si_10_-ncr; (**c**) Al_74_Ni_15_Si_9_Cu_2_-am; and (**d**) Al_74_Ni_15_Si_9_Cu_2_-ufcr.

The corrosion products formed during the immersion test on the surface of the tested amorphous and crystalline ribbons at both temperatures were observed using SEM (Figure 2) and analysed via XRD (Figure 3). After a prolonged immersion test in 3.5% NaCl, the surface of the amorphous alloys was affected by surface corrosion with clearly visible caverns oriented along the ribbon length. Corrosion products appear relatively uniformly deposited and tightly adhered on the surface (Figure 2a,c). On annealed alloys, the corrosion products have an uneven and loose structure, especially in the case of the copper-containing alloy (Figure 2b,d).

Three amorphous humps are clearly visible on the XRD patterns of the corrosion products of all the amorphous alloys. These humps are situated at around 15, 28 and 40 degrees 2Ɵ and correspond to the main peaks of AlO(OH)-Bohemite (Figure 3a,c). These humps are of higher intensity and are more pronounced in the pattern of the Cu-containing Al_74_Ni_15_Si_9_Cu_2_-am alloy (Figure 3c).

On the XRD pattern of the corrosion products of the amorphous Al_74_Ni_16_Si_10_-am alloy (Figure 3a), released at 25 °C and 50 °C, low-intensity peaks of the crystalline phases nickel chloride hydrate NiCl_2_(H_2_O)_4_ and Al(OH)_3_ are observed together with the amorphous humps. This indicates that the released corrosion products at both test temperatures are a mixture of amorphous and crystalline compounds. A similar structure of the corrosion products has been observed by other researchers also [55,56,57,58].

The diffraction pattern of the products of the amorphous Al_74_Ni_15_Si_9_Cu_2_-am alloy released at 25 °C shows a completely X-ray amorphous structure (Figure 3c). The humps intensity of this alloy, compared with the XRD pattern of the Al_74_Ni_16_Si_10_-am alloy, are greater, indicating a greater amount of released amorphous products. Single peaks with a low intensity of the crystal phases CuO and Cu_2_O are observed on the XRD pattern at 50 °C (Figure 3c). Based on these features of the diffractogram, we assume that the structure of the released products at both temperatures is mostly X-ray amorphous.

On the XRD patterns of the corrosion products, released at 25 °C from the nanocrystalline Al_74_Ni_16_Si_10-_ncr and the ultra-fine crystalline Al_74_Ni_15_Si_9_Cu_2_-ufcr alloys, only great intensity NaCl peaks (residues from the 3.5% NaCl solution) are identified. On the XRD patterns at 50 °C of both alloys, amorphous humps of AlO(OH) are visible around 2Ɵ (10–25), (25–35), and (35–48), (Figure 3b,d), and the humps in the copper-containing alloy Al_74_Ni_15_Si_9_Cu_2_-ufcr again are more intense. Based on the form of the XRD patterns, we conclude that the microstructure of the released corrosion products from the both the Al_74_Ni_16_Si_10_-ncr and Al_74_Ni_15_Si_9_Cu_2_-ufcr alloys is a mixture of an X-ray amorphous matrix with singe crystalline phases.

### 3.2. Electrochemical Corrosion Tests

**Figure 4:**
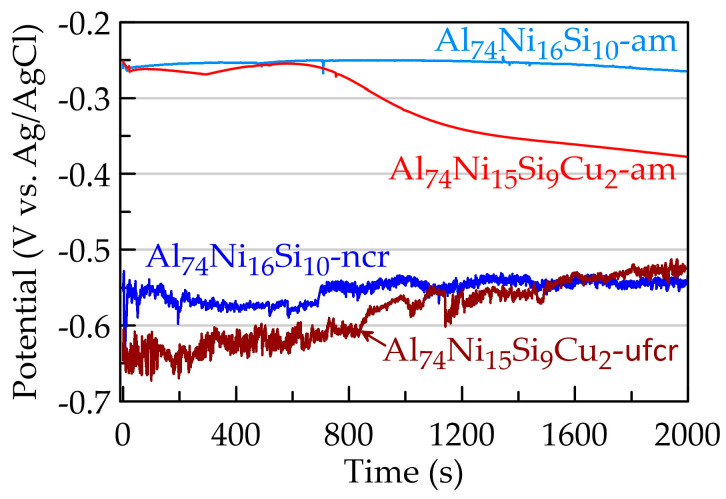
Dependence of open circuit potential on time.

Figure 4 shows the dependences of the change in the open circuit potential (OCP) in contact with 3.5% NaCl. It can be seen that during the first 20 min, the values of OCP for the amorphous alloys are close, but with longer immersion in the chloride media, the potential of the alloy Al_74_Ni_15_Si_9_Cu_2_-am shifts to the negative direction. Negative polarisation is most often associated with accelerating metal dissolution. The values of OCP for the annealed alloys are about 300 mV more negative than those of their amorphous analogues. Moreover, for both the crystalline alloys, the potentials are unstable and continuous fluctuations are observed on the curve. Initially, the amplitude of these oscillations exceeds 50 mV, but gradually decreases to 10 mV.

**Figure 5:**
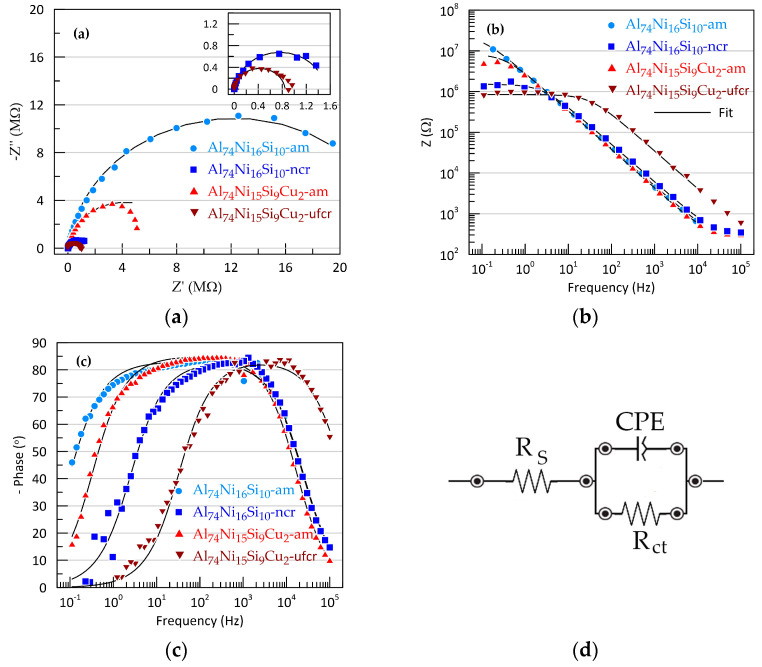
EIS results for Sb-Cu layers: (**a**) Nyquist’s plots, (**b**) Bode impedance plots, (**c**) Bode phase angle plots and (**d**) equivalent circuit used to fit EIS data.

Electrochemical impedance spectroscopy provides additional information on the behaviour of alloys under OCP. The Nyquist plot shows incomplete semicircles typical of passive metal systems. Semicircle response is due to the charge transfer reaction. An increase in semicircle diameter corresponds to an increase in charge transfer and hence the corrosion resistance. At high frequencies, Bode impedance plots are in straight line parallel to the *x*-axis and is a result of the frequency independence of resistance. The intersection of the dependences with the ordinate axis of the impedance module is usually associated with the solution resistance *R*_s_, which dominates in the high-frequency range. In the low-frequency region, the charge transfer process (the electrochemical reaction) dominates. It can be seen from Figure 5b that the amorphous alloys have the highest impedance at 0.1 Hz, while their crystalline analogous are about one order of magnitude lower.

These observations are also confirmed by the Bode phase angle plots (Figure 5c), which have a bell shape. At both very high and very low frequencies, the phase angle deviation tends to be 0, indicating the resistive behaviour of the circuit. In the middle frequency range, the dependences for all tested alloys show a wide plateau, not reaching −90°. The shift in the Bode magnitude maxima to higher frequencies in crystalline alloys indicates an expansion of the frequency range where the charge transfer of corrosion reaction dominates, and charging of the electric double layer takes place at increasingly higher frequencies.

The equivalent circuit model presented in Figure 5d was used to interpret and analyse the EIS data. This model describes processes at the metal–electrolyte interface and electron transfer. The electrical circuit consists of a series-connected *R_s_* (solution resistance) and a block of parallel *R_ct_* (charge transfer resistance) and a constant phase element CPE corresponding to the capacitance of electrochemical double. The CPE is used instead of a pure capacitor since the phase angle in the middle frequencies region does not reach 90° (Figure 5c) as well as an imperfect semicircle in the Nyquist plots. The deviation from the ideal capacitor behaviour is expressed through the constant *n*. However, the recorded deviations are relatively weak, since the value of *n* is above 0.92.

The resulting fits based on this circuit are represented as black lines on the Nyquist and Bode plots (Figure 5).

The values of the parameters of the equivalent circuit for the tested alloys are summarised in Table 3. The EIS data show that while the R_S_ values for all tested alloys remain relatively close, the charge transfer resistance and CPE decrease with the addition of copper and with the transition from the amorphous to the crystalline structure.

**Figure 6:**
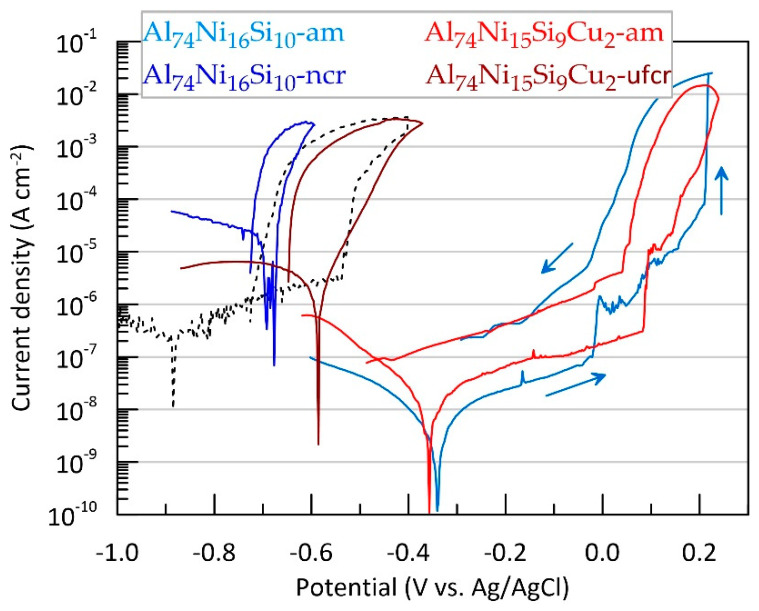
Polarisation dependences of alloys Al_74_Ni_16_Si_10_ and Al_74_Ni_15_Si_9_Cu_2_ in amorphous and crystalline state and of pure aluminium (black dotted line). Arrows indicate the progress of the potential scan.

The corrosion behaviour of the Al-Ni-Si-(Cu) alloys was also tested via the cyclic potentiodynamic polarisation method. Typical example of the measurements is presented in Figure 6. Characteristic corrosion electrochemical parameters such as corrosion potential (*E*_corr_), corrosion current density (*J*_corr_) and pitting potential (*E*_pitt_) were determined from the polarisation dependences. The average values of these parameters are presented in Table 4.

The values of *E_corr_* and *J_corr_* for the two amorphous alloys are close. The more negative value of *E_corr_* and the lower value of Jcorr for the copper-containing alloy can be considered insignificant. A similar trend was also reported by Setiady and Soegijono [38]. The more negative average Ecorr values compared to OCP measured immediately before the polarisation tests showed that the low value of cathodic polarisation and the short time of its application nevertheless negatively polarised the alloys.

The average *E*_corr_ values for Al_74_Ni_15_Si_9_Cu_2_ alloy are about 20 mV more negative and the values for *J*_corr_ deviate over a wide range of more than one order of magnitude (Table 4). Furthermore, it can be noted that the corrosion rate of the amorphous alloys is two orders of magnitude lower and the corrosion potential is more than 500 mV more positive compared to the corresponding values obtained for high-purity polycrystalline aluminium (Figure 6, black dotted line).

The annealing of the alloys leads to a significant deterioration in the corrosion resistance, manifested both in a deviation of *E*_corr_ in the negative direction (with an average of about 340 mV for Al_74_Ni_16_Si_10_ and 200 mV for Al_74_Ni_15_Si_9_Cu_2_) and in an increase in *J*_corr_ (with an average of about three and two orders of magnitude for Al_74_Ni_16_Si_10_ and Al_74_Ni_15_Si_9_Cu_2_, respectively). The lowest corrosion resistance is demonstrated by the annealed copper-free alloy (Table 4).

The anodic branch of the polarisation curves of the amorphous alloys shows diffusion-limited anodic process, which is typical for the presence of passive layers (Figure 6). Upon reaching a certain critical potential called the pitting potential (*E*_pitt_), the anodic current density increases sharply. The latter is an indication of the development of pitting corrosion, typical for aluminium alloys under anodic polarisation in a chloride media. The registered slight fluctuations on the anodic branch of the polarisation dependence before reaching the *E*_pitt_ can be associated with the development of metastable pits which are repassivated immediately after their nucleation. When additional anodic polarisation exceeds *E*_pitt_, some of the pits grow steadily, resulting in a sharp increase in current.

The pitting potential of Al_74_Ni_15_Si_9_Cu_2_-am alloy is more positive by about 100 mV on average compared to Al_74_Ni_16_Si_10_-am. This shows that the presence of 2% copper broadens the passive state. These results are similar to the ones reported by Muller and Galvele for aluminium alloys containing less than 5% copper [59]. According to these authors, the CuAl_2_ phase formation during heating leads to copper depletion of grain boundaries, an increased susceptibility to intercrystalline and pitting corrosion around this phase. These authors also reported Cu enrichment in the interior of the pits.

Scanning the potential in the reverse direction reveals the local corrosion development, characterised by rapid dissolution of limited active areas, which raises the anodic current density to several orders of magnitude higher than that of the forward branch. It is noteworthy that for both amorphous alloys partial repassivation is observed at potentials close to *E*_pitt_. After this value, the reverse branch current remains only about an order of magnitude higher than that of the forward branch, and the polarisation curve maintains a relatively small slope until crossing the cathode branch.

In the case of the annealed alloys, the anodic polarisation does not lead to diffusion limitation of the current; they are not passive, but actively dissolve in the chloride medium.

## 4. Discussions

The initiation and evolution of corrosion processes in metallic materials depend on the chemical composition and the microstructure of the alloys, the chemical composition and temperature of the corrosion environment and on the interaction of these factors.

The results of our immersion tests showed that the corrosion rate of the copper-containing Al_74_Ni_15_Si_9_Cu_2_ alloy both in amorphous and crystalline state was higher than that of the corresponding copper-free alloys. The probable reason for this behaviour in crystalline state is the presence of (Ni,Si,Cu)-containing phases of various composition and size in the Al_74_Ni_15_Si_9_Cu_2_-ncr alloy [47], but this cannot explain the behaviour of the amorphous alloy.

**Figure 7:**
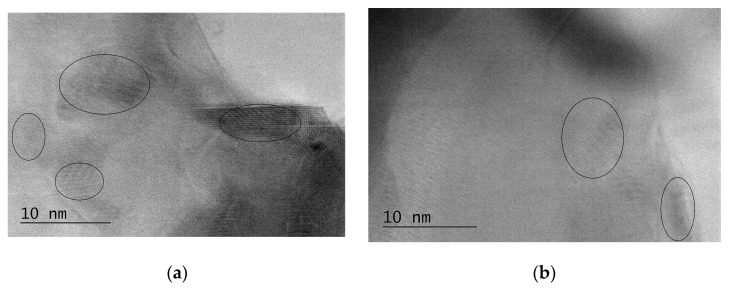
Microstructure of the amorphous Al_74_Ni_16_Si_10_–am (**a**) and Al_74_Ni_15_Si_9_Cu_2_–am (**b**) alloys, HRTEM. Amorphous matrix with clusters of Ni, Si/Cu atoms.

In our previous research, we confirmed via TEM observations the amorphous microstructures of both Al_74_Ni_16_Si_10_ and Al_74_Ni_15_Si_9_Cu_2_ rapidly solidified alloys. A typical amorphous structure uniform matrix with well-defined diffraction halo was registered on the diffractograms of both alloys. Small white spots randomly scattered in the dark uniform matrix were observed on the TEM images of Al_74_Ni_15_Si_9_Cu_2_ [50]. We performed additional high resolution HRTEM observations and found regions corresponding to the white spots in both alloys, but in much larger number in Al_74_Ni_15_Si_9_Cu_2_ (Figure 7). These regions showed a specific striation contrast which made us to believe that they were clusters of atoms of the dissolved alloying elements, probably nickel, silicon and/or copper. No grain boundaries bordered the striated regions indicated that the clusters were formed during rapid solidification but did not succeed to combine into crystal grains. Therefore, we concluded that the microstructures of both rapidly solidified alloys consists of an X-ray amorphous matrix with single clusters of atoms of alloying elements, probably Ni and Si in Al_74_Ni_16_Si_10_, and a large number of clusters, probably Ni, Si and/or Cu in Al_74_Ni_15_Si_9_Cu_2_. The clusters act as microcathodes to the anodic aluminium matrix and form microgalvanic cells that are potential areas for corrosion development [60]. The larger number of clusters in Al_74_Ni_15_Si_9_Cu_2_-am is the reason for the higher corrosion rate of this alloy compared to Al_74_Ni_16_Si_10_-am in long-term corrosion tests.

When comparing the results obtained via gravimetric and electrochemical tests at 25 °C, some discrepancies between the two methods appear. The immersion test results show a lower corrosion rate CR_25_ of Al_74_Ni_16_Si_10_-ncr compared to Al_74_Ni_15_Si_9_Cu_2_-ufcr, while the potentiodynamic polarization test shows a negligible difference in corrosion potential. A probable reason for this discrepancy can be found in the morphological features of the rapidly solidified ribbons and the specificity of the used corrosion test methods.

The two sides of the ribbons obtained via the CBMS method have different roughness (Figure 1). The surface which has been in contact with the surface of the cooling copper disk is rather rough (Figure 1a)—there are hollows (the darker areas) and ridges (the lighter areas) on it. These places are additional potential sites for corrosion initiation. During the gravimetric immersion tests, the rough side of the samples was not isolated in order to ensure a larger active surface area. After the enhanced corrosion initiation occurred due to surface roughness, the cathodic areas on the rough surface are increasingly revealed during the prolonged immersion in the corrosive environment (360 h), and this accelerates the general dissolution of the anodic Al-matrix.

We can conclude that the absence of a significant influence of copper on the electrochemical test results of the amorphous alloys is a result of both the smoother surface and the short immersion time in the chloride medium. Over time, the differences between the two sides of the ribbons decrease and the negative effect of the copper-rich cathodic phases accelerates the corrosion process. This is clearly observed from both the OCP dependences and the SEM observations. After annealing, the corrosion resistance of both alloys decreases due to an increase in surface heterogeneity.

The prolonged immersion tests involve the rough side also and this results in higher calculated values of the corrosion rate. Keeping in mind the significantly more positive potential of copper, it can be expected that the participation of copper, in spite of the smaller content of Cu than Ni in the alloy, will have a stronger negative effect on the corrosion of microgalvanic cells during long-term treatment compared to those based on nickel.

Summarizing the results of both the corrosion tests and the TEM and SEM observations, we can conclude that in the amorphous Al-Ni-Si and Al-Ni-Si-Cu alloys in 3.5% NaCl solution, general surface corrosion is mainly observed, which is accelerated by the anodic dissolution of the aluminium matrix caused by the microgalvanic cathodes (polymetallic clusters). Corrosion damage is visibly better expressed in the copper-containing amorphous alloy. Due to the greater total area of the cathodic phases in crystalline alloys, corrosion develops primarily through a galvanic mechanism.

## 5. Conclusions

The addition of 2 at.% copper to the amorphous Al_74_Ni_16_Si_10_ base alloy improves the pitting corrosion resistance of the amorphous Al_74_Ni_15_Si_9_Cu_2_, shifting the pitting potential by about 100 mV in the positive direction, but does not significantly affect the corrosion current and potential in the duration when short-term polarisation tests were performed. However, in long-term immersion tests, the copper-containing alloy Al_74_Ni_15_Si_9_Cu_2_ demonstrates 2.6 times lower general corrosion resistance both in amorphous and crystalline states and at both temperatures.

The amorphous Al_74_Ni_16_Si_10_ and Al_74_Ni_15_Si_9_Cu_2_ alloys have better corrosion resistance compared to their crystalline analogues. After crystallisation, the corrosion rate of both the alloys increases by about 3 times at a test temperature of 25 °C and by about 8 times at a temperature of 50 °C. The corrosion rates of the crystal alloys Al_74_Ni_16_Si_10_ and Al_74_Ni_15_Si_9_Cu_2_ increase 5 and 2 times at test temperatures 25 °C and 50 °C, respectively.

The thin film of corrosion products released from the tested amorphous and crystallised copper-containing Al_74_Ni_15_Si_9_Cu_2_ alloys is completely X-ray amorphous, and the film released from the copper-free alloy Al_74_Ni_16_Si_10_ is mostly X-ray amorphous with single peaks of crystalline phases.

## Figures and Tables

**Table 1:** Chemical composition of the tested Al-Ni-Si and Al-Ni-Si-Cu alloys.

Designation of the Alloy	Al [at.%]	Ni [at.%]	Si [at.%]	Cu [at.%]
Al-Ni-Si	73.50	16.08	10.42	-
Al-Ni-Si-Cu	73.97	14.94	8.76	2.33

**Table 2:** Corrosion rate of Al_74_Ni_16_Si_10_ and Al_74_Ni_15_Si_9_Cu_2_ alloys in amorphous and crystalline states.

Corrosion Rate CR [g m^−2^h^−1^]						
Designation of the Alloy	CR_25_ T = 25 °C	CR_50_ T = 50 °C	A	B	C	D
Al_74_Ni_16_Si_10_ am	0.35 × 10^−2^	0.67 × 10^−2^	2.9	8.4	1.9	-
Al_74_Ni_16_Si_10_ ncr	1.02 × 10^−2^	5.63 × 10^−2^			-	5.5
Al_74_Ni_15_Si_9_Cu_2_ am	0.92 × 10^−2^	0.95 × 10^−2^	3.3	7.7	1	-
Al_74_Ni_15_Si_9_Cu_2_ ufcr	3.07 × 10^−2^	7.3 × 10^−2^			-	2.4

**Table 3:** Equivalent electrical circuit parameters for Al-Ni-Si-(Cu) alloys at OCP.

Designation of the Alloy	*R*_S_ (Ω)	*R*_ct_ (MΩ)	CPE (µΩ.s^n^)	*n*
**Al_74_Ni_16_Si_10_-am**	256.0	24.51	0.064	0.924
**Al_74_Ni_15_Si_9_Cu_2_-am**	295.0	8.36	0.059	0.945
**Al_74_Ni_16_Si_10_-ncr**	310.7	1.52	0.051	0.925
**Al_74_Ni_15_Si_9_Cu_2_-ufcr**	248.9	0.85	0.008	0.931

**Table 4:** Corrosion parameters extracted from the polarisation dependences of amorphous and crystalline Al-Ni-Si-(Cu) alloys.

Designation of the Alloy	*E*_corr_ (V)	*J*_corr_ (µA/cm^2^)	*E*_pitt_ (V)
**Al_74_Ni_16_Si_10_-am**	−0.326 ± 0.043	0.009 ± 0.002	−0.029 ± 0.050
**Al_74_Ni_15_Si_9_Cu_2_-am**	−0.349 ± 0.044	0.005 ± 0.003	0.068 ± 0.046
**Al_74_Ni_16_Si_10_-ncr**	−0.669 ± 0.010	14.60 ± 4.20	-
**Al_74_Ni_15_Si_9_Cu_2_-ufcr**	−0.557 ± 0.034	0.508 ± 0.077	-

## Data Availability

Not applicable.
